# Fish oil-containing edible films with active film incorporated with extract of *Psidium guajava *leaves: preparation and characterization of double-layered edible film

**DOI:** 10.12688/f1000research.153383.2

**Published:** 2024-10-30

**Authors:** Aji Sukoco, Yukihiro Yamamoto, Hiroyuki Harada, Atsushi Hashimoto, Tomoyuki Yoshino

**Affiliations:** 1 Study Program of Agricultural Product Technology, Universitas Jember, Jember, East Java, 68121, Indonesia; 2 Graduate School of Comprehensive Scientific Research, Prefectural University of Hiroshima, Shobara, Hiroshima, 727 0023, Japan; 3 Faculty of Bioresource Sciences, Prefectural University of Hiroshima, Nanatsuka-cho 5562, Shobara, Hiroshima, 727 0023, Japan

**Keywords:** Antibacterial, antioxidant, double-layer, edible film, fish oil, guava leaf extract, gum Arabic, zein

## Abstract

The utilization of zein and gum arabic has grown in an attempt to formulate wall materials based on protein–polysaccharide complexes. This mixture provides a versatile delivery system for hydrophilic (guava leaf extract, GLE) or lipophilic (fish oil, FO) bioactive compounds, and it can be used as an edible film-forming polymer. This study was undertaken to characterize FO-containing edible films that were double-layered with a film containing GLE. Modified zein and gum arabic solutions (MG complex) were mixed at a ratio of 1:1.5 (v/v), adjusted to pH 5, added with glycerol (20% of the complex) and FO (5% of the complex), and finally adjusted to pH 5. This was prepared as the bottom/lower layer. The upper/active layer was prepared by mixing MG complex, glycerol, and GLE (1, 3, and 5% w/v of the complex). The total phenolic and flavonoid contents in GLE were 15.81 mg GAE/g extract and 6.99 mg QE/g extract, respectively. The IC50 of the DPPH radical scavenging activity of GLE was 26.86 ppm with antibacterial activity against *Bacillus subtilis* and *Escherichia coli* of 9.83 and 12.55 mm. The total plate counts of double-layered films containing GLE were retained below 3 log CFU/g during 28-day storage. The peroxide values of these films were dimmed for no more than 9.08 meq/kg sample on day 28 of storage. Thickness (872.00-971.67 μm), water vapor transmission rate (12.99-17.04 g/m ^2^/day), tensile strength (1.56-2.02 kPa), elongation at break (61.53-75.41%), glass transition (52.74-57.50°C), melting peak (131.59-142.35°C), inhibition against *B. subtilis* (33.67-40.58 mm), and inhibition against *E. coli* (2.05-9.04 mm) were obtained by double-layered films. GLE can be successfully incorporated into the active layer of a double-layer film to improve its characteristics while significantly slowing down the microbial contamination and oxidation rate. MG complex and FO can also contribute to the performance of the edible film.

## Introduction

Fish oil (FO) is a precious source of bioactive lipids such as omega-3 fatty acids, which can improve numerous health functions. Previous studies have demonstrated the positive impact of FO consumption on the health and brain development of children. ^1^^–^^3^ In the context of alleviating the risk of disease, FO has been investigated to mitigate fracture, cardiovascular, colorectal, prostate, and chronic kidney disorders in people over the age of 50. ^4^^–^^8^ Therefore, a wide utilization of FO is not limited to dietary supplements. Enrichment of FO in solid to liquid food products, such as dark chocolate, ^9^ cookies, ^10^ granola bars, ^11^ sausage, ^12^ milk chocolate, ^13^ and yogurt ^14^ has also been attempted. However, owing to the high content of omega-3 fatty acids, the oxidative stability of FO in food products can be compromised. Despite encapsulation-stabilized FO, oxidation remains a challenge because the direct incorporation of encapsulated FO may affect the stability of its wall materials during food processing, allowing interaction with food ingredients and external factors. The latter can exaggerate the simultaneous oxidation reaction that leads to the rejection of food products owing to off-flavors.

An alternative to enriching food with FO has been established in previous studies through the preparation of edible films containing FO alone or in combination with essential oils. ^15^^–^^18^ They found that FO alone could increase the elongation of the film, and the combination of essential oils dampened the oxidation rate. Edible film is the outer part of the food matrix, and air is the most influential factor in the oxidation process that directly interacts with the film. Thus, a double-layered film can be proposed to minimize the oxidation of FO in the matrix of the edible film. A multilayer design can enhance the functionality of the packaging outstrip mono-layered design. ^19^ In this study, the first (lower) layer was made from edible film solution containing FO only, and the edible film solution for the second (upper) layer was incorporated with active compounds only.

In addition to essential oils, plant extracts can be used to strengthen the upper/active layer. Several previous studies have investigated the combination of FO and apple peel extract, ^20^ cashew leaf extract, ^21^ and green tea extract. ^22^ Among other plant-based sources of active compounds, guava ( *Psidium guajava*) leaves have been widely exploited as a popular natural remedy in traditional medicine. Secondary metabolites derived from leaves provide excellent antioxidant and antimicrobial activities, ^23^ making them a valuable (high bioactivity, abundance, and low price) part of agricultural waste. Thus, guava leaf extract (GLE) is a suitable candidate for protecting edible films rich in FO.

The selection of film-forming polymers is also a crucial step for the successful implementation of this idea. Protein-polysaccharide complexes have been developed for the preparation of edible films and the encapsulation of FO. Gum arabic (GA) can be used as a polysaccharide in edible films because it is safe to use as an emulsifying and stabilizing agent in food processing. They are also abundantly available and inexpensive. Because poor water vapor resistance and high brittleness are the main shortcomings of GA-based film, ^24^ it requires an additional ingredient that can reinforce their barrier and mechanical properties. Zein is a plant protein derived from corn that has good biocompatibility and biodegradability. Zein contains more than 50% hydrophobic amino acids, ^25^ it is useful to improve the characteristics of edible films. Simultaneously, FO can be stabilized by the hydrophobic amino acids of zein. Peppermint oil, medium-chain triglyceride oil, and resveratrol have been successfully protected using a wall material based on a zein-GA mixture. ^26^^–^^28^ We used glycerol and sodium dodecyl sulfate (SDS) to prevent the aggregation of zein particles. To the best of our knowledge, no reports have documented the combination of zein and GA as polymers for the preparation of double-layer edible films, in which one layer contains FO and the other contains GLE. Therefore, this study was performed to evaluate the physical, mechanical, microstructural, thermal, microbiological, and oxidative properties of FO-containing edible films double-layered with a film containing GLE.

## Methods

### Materials

FO was purchased from Tama Biochemical Co. Ltd. (Tokyo, Japan). Guava leaves were collected from the tertiary and secondary branches of guava trees aged four years. The guava trees were cultivated by local farmers in Banyuwangi, Indonesia. Pure zein powder, GA powder, glycerol, sodium dodecyl sulfate, sodium carbonate, aluminum nitrate, potassium acetate, calcium chloride, potassium chloride, potassium iodide, gallic acid monohydrate, quercetin dihydrate, ascorbic acid, sodium thiosulfate, isooctane, isopropanol, methanol, and acetic acid were obtained from Fujifilm Wako (Osaka, Japan). 1,1-Diphenyl-2-picrylhydrazyl (DPPH) was purchased from TCI Chemical Trading Co., Ltd. (Tokyo, Japan). Folin-Ciocalteu reagent was acquired from Kanto Chemical Co. Inc. (Tokyo, Japan). Tryptic soy agar (TSA) (Bacto™, Maryland, USA), peptone (Bacto™, Maryland, USA), 6 mm paper discs (ADVANTEC, Tokyo, Japan), *Escherichia coli* NBRC 3972 (Tokyo, Japan), and *Bacillus subtilis* JCM 20094 (Ibaraki, Japan) were used for microbiological analysis. Filter paper 5C (185 mm) (ADVANTEC, Tokyo, Japan) and membrane filter 1.0 μm (Millipore, Ireland) were used for the extraction process. Filter paper 2 (90 mm) (ADVANTEC, Tokyo, Japan) was used for the filtration process in the analysis of peroxide values. Ultrapure water (Type 1) was used in this study.

### Preparation of guava leaf extract

In this study, GLE was prepared using water as a solvent. Fresh leaves were washed and dried in an oven at 60°C for approximately 48 h. The leaves were then ground and sieved through a 60-mesh sieve to obtain a fine guava leaf powder (moisture content of 10.13%). A weighed powder (50 g) was dissolved in 500 mL of water. The mixture was stirred at 1100 rpm for 60 min at 70°C. It was then filtered using filter paper (185 mm). The supernatant was subsequently filtered using a vacuum filtration apparatus (SIBATA, Japan) equipped with a membrane filter 1.0 μm which removed fine impurities and large-sized bacteria. Water was evaporated for 3 h at 60°C using a rotary evaporator to concentrate the extract from 1 to 25 °Brix. The GLE was stored in a refrigerator (4°C). ^29^

### Analysis of bioactive compounds of GLE

The total phenolic content was determined using the Folin-Ciocalteu method, as described by Kim *et al*. ^30^ with modifications. Serial concentrations (10, 20, 30, and 40 μg/mL) of gallic acid standard were prepared from a stock standard solution of 1000 μg/mL. A volume (500 μL) of each concentration was added to 500 μL of Folin–Ciocalteu phenol reagent. The mixture was then shaken and incubated for 8 min. Subsequently, 4 mL 7.5% sodium carbonate was added to the mixture and vigorously shaken using a vortex. Approximately 10 mL of water was then added. Incubation was performed for 60 min at 24±2°C in the dark. Absorbance was measured at 730 nm using a UV-Vis spectrophotometer (Hitachi U-2900, Japan). A calibration curve was established based on the absorbance against serial concentrations. This procedure was also used to measure the sample (extract) solution at a concentration of 1000 μg/mL. The total phenolic content was expressed as milligrams of gallic acid equivalents per gram of extract (mg GAE/g extract).

The total flavonoid content was analyzed according to Otsuka *et al*. ^31^ with slight modifications. A quercetin standard solution (1000 μg/mL) was prepared to obtain serial concentrations (2.5, 5, 10, 20, 40, and 80 μg/mL) of the standard. Water (1.5 mL) was transferred to 500 μL of each concentration, and 100 μL of 10% aluminum nitrate was subsequently added. A volume (100 μL) of 1M potassium acetate was added to the mixture. A new mixture was incubated in the dark for 40 min at 24±2°C. The absorbance of 1 mL of the mixture was measured at 450 nm using a UV-Vis spectrophotometer (Hitachi U-2900, Japan). Measurement of the total flavonoid content of the extract (1000 μg/mL) was performed using the same procedure. The results were expressed as milligrams of quercetin equivalents per gram of extract (mg QE/g extract).

### Analysis of antioxidant activity of GLE

DPPH radical scavenging activity was assayed using the method described by Brand-Williams *et al*. ^32^ with modifications. DPPH solution (0.1 mM) was prepared using methanol. The extract was prepared at concentrations of 1000 μg/mL and serial concentrations (20, 60, 100, 140, and 180 μg/mL) were then made. Two milliliters of each concentration were mixed with 2 mL of DPPH solution and incubated for 30 min in the dark at 24±2°C. The absorbance was then measured at 517 nm using a UV-Vis spectrophotometer (Hitachi U-2900, Japan). Ascorbic acid (AA) was used as the standard at concentrations of 2, 4, 6, 8, and 10 μg/mL. Antioxidant activity was expressed as the IC _50_ value (ppm). It was calculated from the % inhibition curve, and the AA standard was used for comparison.

### Analysis of diameter of inhibition zone

An agar disc diffusion test was conducted to determine the antibacterial activities of GLE and edible films against *B. subtilis* and *E. coli.* Briefly, an aliquot (100 μL) of a suspension of *B. subtilis* (10 ^7^ CFU/mL) or *E. coli* (10 ^6^ CFU/mL) was evenly spread onto solid TSA medium. One hundred microliters of GLE at several concentrations (25, 50, 75, and 100%) were dropped onto a sterile paper disc with a diameter of 6 mm. The discs were allowed to dry for 10 min in a sterile glass Petri dish and then placed on the inoculated media. The edible film samples were cut to a size of 6×6 mm. The dishes were incubated for 48 h at 30°C. The diameter was measured in millimeters using a digital caliper (Niigata Seiki SK DT-150, Japan). ^33^

### Preparation of modified zein-gum arabic complex

A zein solution (5%, w/w) was prepared from pure zein powder in aqueous acetic acid (80%, v/v). ^34^ After stirring for 12 h, acetic acid was evaporated for 4 h using a rotary evaporator. The solution was washed with water to completely remove the acetic acid. The zein dough was then soaked under stirring (600 rpm) in hot (60°C) glycerol, a nonionic surfactant, for 15 min to interact with the hydrophilic amino acid residues of the unfolded zein. Next, the dough was left at room temperature (24±2°C) for 30 min and oven-dried at 60°C for 7 h. The dried dough was then ground for a few seconds and sieved through a 100-mesh sieve. Finally, a fine powder with a moisture content of 6.52% was obtained.

The next step involved an anionic surfactant, SDS solution (3%, w/v), which stabilized the zein proteins through electrostatic interactions between its negatively charged head with arginine, histidine, and lysine. In addition, its hydrophobic tail can interact with hydrophobic amino acids. These two types of surfactants were responsible for the stabilization of the emulsion in the next step. A modified zein solution (5%, w/w) was prepared using fine powder in an SDS solution (3%, w/v). The mixture was stirred at 1000 rpm and 90°C until a homogenous solution was obtained. A GA solution (20% w/w) was also prepared using GA powder in water. To prepare a modified zein-GA (MG) complex at a ratio of 1:1.5, a centrifuged (8250 rpm, 24°C, 10 min) GA solution was slowly added to the modified zein solution while gently stirring for 20 min at 24±2°C. The pH of the complexes was adjusted to 5. Based on our preliminary experiments, this ratio and pH condition were the most stable conditions for preparing FO emulsions.

### Preparation of double-layer edible film

First, the freshly prepared MG complex was slowly added to glycerol (20%, v/v of the complex) under stirring (1000 rpm) at 60°C for 10 min. Subsequently, FO (5% v/v of the complex) was added dropwise to the mixture under the same stirring conditions. FO 5% was chosen based on the preliminary test in our lab. It was revealed that at a concentration of 5%, FO properly improved the characteristics of the edible film in structural, thermal, and oxidative stability aspects, compared to those of FO 10 and 15% (unpublished report). Homogenization (Homogenizer Ultra-Turrax T25 B S1, Malaysia) was carried out at 19000 rpm for 5 min. The emulsion was continuously processed using a bath sonicator (Yamato model 3510 BRANSON, USA) at 20°C for 20 min, and then adjusted to pH 5. A volume (25 mL) of the emulsion was poured into a polystyrene Petri dish (9 cm in diameter) and dried in a conventional oven at 40°C. A single-layer edible film containing FO (MGFO) was obtained after drying for 48 h.

The mixture in the upper layer was immediately poured onto the single/bottom layer to form a double-layer film. The mixture was prepared without FO. The freshly prepared complex, glycerol 20%, and GLE 100% at additional levels of 1, 3, and 5% (w/v of the complex) were mixed and stirred at 1000 rpm for 10 min at 60°C. GLE at an additional level of less than 1% did not significantly improve the oxidative stability and antibacterial activity of FO-containing edible film (unpublished report). There was no homogenization, sonication, or pH adjustment after processing. Approximately 25 mL of the mixture was cast onto the bottom layer and dried for 48 h. The last stage was peeling the double-layer film off the petri dish, and the film was stored in a zip-lock bag at room temperature (24±2°C). The samples were labeled based on the additional level of GLE: MGFO-GLE 1%, MGFO-GLE 3%, and MGFO-GLE 5%.

### Film characterization

**Analysis of moisture content**

Approximately 1 g of double-layer edible film was weighed in the sample pan of a moisture analyzer (AND ML-50, Japan), and the moisture content was reported as % after drying at 105°C.

**Thickness analysis**

The thickness of the double-layer edible film was measured using a Digimatic Micrometer (Mitutoyo IP 65, Japan) at ten randomly selected spots.

**Analysis of water vapor transmission rate**

Vials, diameter and height respectively were 3 and 4.5 cm, were used for this measurement following the protocol of ASTM E96 with slight modifications. ^35^ A weighed (5 g) amount of calcium chloride (RH 2%) was placed in a vial. A double-layer edible film (3 cm in diameter) was fitted to the top of the vial, and its position was maintained using parafilm. The weight of the vial containing calcium chloride and the film was then recorded, and the vial was kept in a desiccator containing a saturated solution of potassium chloride (RH 84%, at 25°C). The weight of the vial was recorded every 4 h for 3 weeks. The weight gain of the vial against time was plotted and the resulting slope was used to calculate the water vapor transmission rate (WVTR). The results were expressed as grams per square meter per day (g/m ^2^/day).

**Analysis of mechanical properties**

Tensile strength and elongation at break (EAB) measurements were performed using a texture analyzer model EZ-SX (Shimadzu, Japan) based on the protocol of ASTM D882-02 with slight modifications. ^36^ A double-layer edible film was prepared in rectangular strips at 1×7 cm and then handled by the grips. The measurement conditions were set to 50 mm, 25 mm/min, and 200 N for the initial grip separation, tensile speed, and load cell, respectively. The tensile strength was reported as kilopascals (kPa) and EAB as a percentage (%).

### Structural analysis

The surface morphology of the double-layer edible film was observed using scanning electron microscopy (SEM) (Miniscope TM4000Plus II Hitachi, Japan). Surface images from the top (100× and 1000× magnification) and bottom (1000× magnification) sides of the films were captured.

### Thermal analysis

Differential scanning calorimetry (DSC) was performed using a DSC instrument (EXSTAR6000 model DSC6100 Hitachi, Japan) and thermogravimetric analysis (TGA) was performed using a TG/DTA instrument (EXSTAR6000 model TG/DTA 6200 Hitachi, Japan). Liquid nitrogen was supplied by a liquid nitrogen auto Supplier JSN-100DP-AS (Japan). The film (5.50-6.50 mg) was weighed in an aluminum pan using an analytical balance (Sartorius CP324S, Germany). After sealing, the pan containing the film and an empty pan (reference) were placed in the DSC or TG/DTA chamber. DSC analysis was performed in the temperature range of -30 to 270°C with the nitrogen flow rate and heating rate set at 50 mL/min and 10°C/min, respectively. The TGA was performed from room temperature to 270°C at a heating rate of 10°C/min.

### Analysis of total plate count

This analysis was performed using a pour-plate method following the protocol of ISO 4833-1. ^37^ A 0.1% peptone solution was prepared as a diluent. A suspension of sample (10 ^−1^) was prepared by adding 1 g of sample (single- or double-layered film) to 9 mL of diluent. The mixture was homogenized using a vortex, and serial dilutions were subsequently made from 10 ^−1^ to 10 ^−3^. From each dilution, 1 mL of the solution was transferred to a sterile disposable petri dish (diameter of 9 cm), followed by the addition of liquid TSA medium. The dishes were incubated for 48 h at 30°C.

### Analysis of peroxide value

The peroxide value (PV) was determined according to the method of Duan *et al*. ^17^ with modifications. Single- and double-layered films (2.5 g) were weighed in sterile disposable centrifuge tubes, and 20 mL of an isooctane-isopropanol (3:1, v/v) mixture ^38^ was added. The tubes were vigorously shaken and centrifuged at 8000 rpm for 10 min at 24°C. Filter paper (90 mm) was used to filter the homogenate. Approximately 0.5 g of the resulting solution was then mixed with 10 mL of isooctane-acetic acid (3:2, v/v) mixture. Approximately 200 μL of a saturated solution of potassium iodide was added to the mixture and gently shaken for 1 min. Subsequently, 80 mL of water was added, and the mixture was titrated with 0.01 N sodium thiosulfate. Automatic titration was performed using an automatic titrator (GT-200 Mitsubishi Chemical Analytech Co., Ltd., Japan).

### Statistical analysis

Data were processed by one-way analysis of variance (ANOVA) using SPSS software (version 16.0). Duncan’s test with a significance level of P < 0.05 was performed to determine differences among the mean values of samples.

## Results and discussion

### Characteristics of GLE

The extraction yield, bioactive compounds, antioxidant activity, and antibacterial activity of GLE are summarized in Table 1. Concentrating the GLE to 25 °Brix gave a yield of approximately 32%, and its pH was close to neutral because the extraction process utilized water solvent. The phenolic and flavonoid contents were obtained at approximately 15.81 mg GAE/g extract and 6.99 mg QE/g extract. These results were lower than the phenolic (24-58 mg GAE/g extract) and flavonoid (75-96 mg QE/g extract) contents of GLE observed in previous studies. ^39^^,^^40^ The differences in the extraction method and conditions make water as a solvent, not the sole factor for this result. Non-conventional extraction techniques, such as microwave- and ultrasound-assisted extraction, have been used to effectively extract bioactive compounds from plant cells. For the conventional extraction method, the use of combination techniques for extraction, such as heating followed by stirring at room temperature for a long time (6 h), maceration and stirring, maceration and heating, and other combinatory models, can produce higher amounts of bioactive compounds. However, our simple conventional extraction method could satisfy the antioxidant activity of GLE, which was categorized as very strong. ^41^ The IC _50_ (4.89 ppm) of ascorbic acid as a standard was about five times lower (greater antioxidant activity) than that of GLE (IC _50_ = 26.86 ppm). In addition, the GLE obtained in this study exhibited antibacterial activity against *B. subtilis* and *E.* *coli* in the inhibition range from weak to strong. The antioxidant and antibacterial activities of GLE were comparable to those of previous reports. ^42^^,^^43^ Non-phenolic phytochemicals may also be responsible for these results; thus, a comprehensive phytochemical analysis of GLE should be performed in future studies. GLE 100% showed a more powerful inhibition against the tested bacteria; therefore, it was selected as the active compound for the upper/active layer of our double-layer films.

**Table 1.** Characteristics of guava leaf extract.

Analysis	Results
Total soluble solids (°Brix)	25.00 ± 0.00
Extraction yield (%)	32.06 ± 2.19
pH	6.81 ± 0.03
Total phenolic content (mg GAE/g extract)	15.81 ± 0.91
Total flavonoid content (mg QE/g extract)	6.99 ± 0.22
IC _50_ of DPPH scavenging activity (ppm)	26.86 ± 2.46
DIZ (mm) of GLE against *B.* *subtilis* at:	
GLE 0% (control)	0.00 ± 0.00 (no inhibition)
GLE 25%	2.14 ± 0.86 (weak)
GLE 50%	3.52 ± 0.91 (weak)
GLE 75%	6.51 ± 0.89 (moderate)
GLE 100%	9.83 ± 0.29 (moderate)
DIZ (mm) of GLE against *E.* *coli* at:	
GLE 0% (control)	0.00 ± 0.00 (no inhibition)
GLE 25%	5.70 ± 0.23 (weak)
GLE 50%	7.74 ± 0.14 (moderate)
GLE 75%	9.79 ± 0.60 (moderate)
GLE 100%	12.55 ± 0.63 (strong)

DIZ: Diameter of inhibition zone; GLE: Guava leaf extract. Values are presented as the mean ± standard deviation (n = 3).

### Physical and mechanical properties of double-layer edible film

Table 2 shows the significant impact of additional levels of GLE on the thickness and WVTR of the films (P < 0.05). The results indicated that a higher addition of GLE contributed to thicker films of approximately 50 μm thickness. The accumulation of higher GLE in the film matrix might have increased its solid content. However, an increase in film thickness had a detrimental effect on the WVTR of the film. The increase in the WVTR value can be attributed to the significant increase in the moisture content of the film (P < 0.05). Since the extraction process employed a water solvent, it is obvious that GLE is rich in hydrophilic compounds, thereby increasing the hydrophilicity of the film surface. Adding GLE 5% resulted in the highest thickness (971.67 μm) but barrier properties (WVTR = 17.04 g/m ^2^/day) against moisture weakened. This result agreed with a previous study that reported that gelatin-based edible films containing GLE alone had the highest thickness but lower moisture resistance. ^44^ Additionally, the lower layer without GLE can be responsible for the reinforced water vapor barrier with respect to the hydrophobicity of zein and FO. Therefore, further characterizations of the physical properties of MGFO are needed to gain a comprehensive understanding of single- and double-layered edible films.

**Table 2.** Moisture content, physical and mechanical properties of double-layer films containing GLE at different addition levels.

Film type	Moisture content (%)	Thickness (μm)	WVTR (g/m ^2^/day)	Tensile strength (kPa)	EAB (%)
MGFO-GLE 1%	3.21 ± 0.20 ^c^	872.00 ± 5.24 ^c^	12.99 ± 0.66 ^c^	2.02 ± 0.30 ^a^	61.53 ± 14.22 ^a^
MGFO-GLE 3%	3.90 ± 0.21 ^b^	922.11 ± 20.19 ^b^	15.35 ± 0.39 ^b^	1.74 ± 0.13 ^a^	65.87 ± 6.69 ^a^
MGFO-GLE 5%	4.54 ± 0.08 ^a^	971.67 ± 18.15 ^a^	17.04 ± 0.47 ^a^	1.56 ± 0.23 ^a^	75.41 ± 15.29 ^a^

WVTR (water vapor transmission rate), EAB (elongation at break). MGFO-GLE 1%: FO-containing edible films double layered with a film containing GLE 1%, MGFO-GLE 3%: FO-containing edible films double-layered with a film containing GLE 3%, and MGFO-GLE 5%: FO-containing edible films double layered with a film containing GLE 5%. Values are presented as the mean ± standard deviation (n = 3). Values with different lowercase superscripts in the same column indicate significant differences (P < 0.05).

In contrast, the addition of GLE did not significantly influence the mechanical properties of the double-layer films (P > 0.05), as shown in Table 2. By adding a lower level of GLE, the tensile strength of the film tended to increase, but the EAB constantly decreased. In this regard, there are fewer plasticizing effects of phenolic compounds of GLE and water in the MGFO-GLE 1%. ^45^^,^^46^ The lowest addition level of GLE may result in a less hydrophilic film because the massive interaction of phenolic compounds and water molecules is minimized. Consequently, a higher strength (2.02 kPa) was required to break a thinner (872 μm) film. Instead, thicker films of MGFO-GLE 3% and MGFO-GLE 5% were less rigid and had higher extensibility due to stronger plasticizing effects and higher moisture content. The interaction between phenolic compounds and biopolymers may also have restricted the formation of intermolecular hydrogen bonds to exert a structural relaxation of the film. Previous findings found a lower EAB (less than 15%) with a higher tensile strength (less than 0.8 MPa) for FO-containing edible film enriched with rosemary and oregano essential oils. ^17^ The types of natural active substances and biopolymers can impart distinctive effects on the mechanical aspect of edible films due to chemical bonds and structural formation between them. Nevertheless, the thickness of the double-layer film obtained in this study should be reduced by determining the suitable volume of the film-forming mixture for the bottom and upper layers.

### Morphology of double-layer edible film

**Figure 1.**
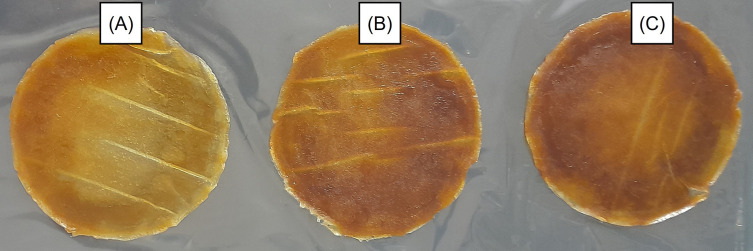
Photographs of double-layer films containing GLE 1% (A), 3% (B), and 5% (C).

**Figure 2.**
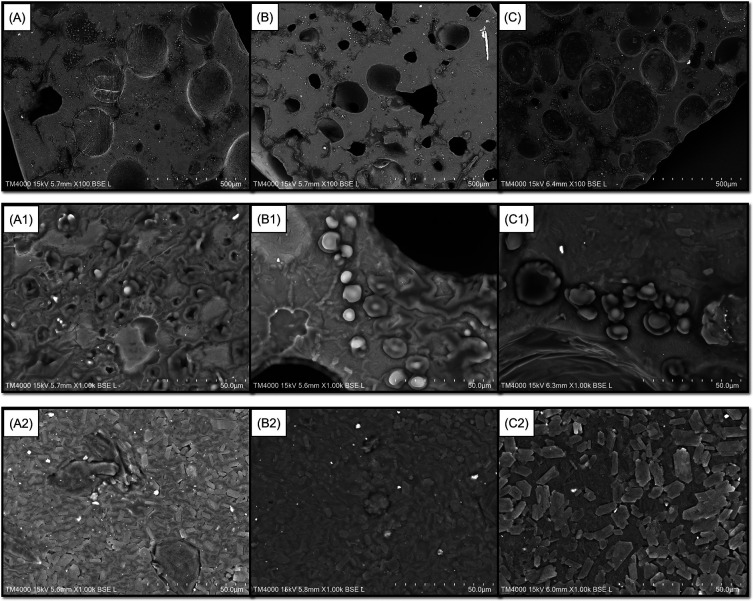
Top (A, B, C, A1, B1, C1) and bottom (A2, B2, C2) sides of films. Top sides of films incorporated with GLE 1% (A), 3% (B), and 5% (C) (SEM images for 100× magnification). Top sides of films incorporated with GLE 1% (A1), 3% (B1), and 5% (C1) (SEM images for 1000× magnification). Bottom sides of films incorporated with GLE 1% (A2), 3% (B2), and 5% (C2) (SEM images for 1000× magnification).

Photographs of the double-layer films are shown in Figure 1. Darker films are shown in Figure 1B and 1C with GLE levels of 3 and 5%, respectively, while the addition level of GLE 1% produced a slightly lighter appearance ( Figure 1A). Furthermore, the latter also presented a film with fewer cavities and troughs on its surface when magnified 100 times using SEM ( Figure 2A). The incorporation of 3 and 5% GLE created more cavities and troughs, making the film surfaces more uneven ( Figure 2B and 2C, respectively). This observation confirmed the WVTR results because the double-layer films incorporated with higher levels of GLE (3 and 5%) showed higher WVTR values for MGFO-GLE 3% and MGFO-GLE 5% ( Table 2). A similar phenomenon was also observed in previous reports dealing with the incorporation of GLE, ^44^ tea polyphenol, ^47^ and seed extract of *Syzygium cumini.* ^48^ The porous surface directly permits water vapor transfer from the external or internal surroundings through the film matrix. Additionally, uneven surfaces with large cavities and troughs have made MGFO-GLE 3% and MGFO-GLE 5% less resistant (easy to break) to applying tensile strength. It was indicated by lower tensile strength values but higher EAB values, at once, confirming that they were inversely proportional relationships.

Perhaps the infiltration of FO droplets into the upper/active layer during the drying process of the film disturbed the integrity of the upper film-forming mixture, which was more hydrophilic. With respect to the ability of the MG complex to act as a wall material, such a lipophilic compound will be protected inside it. Hence, there is a dual function of the MG complex in the upper-layer matrix. On one hand, it functions to construct an interaction with GLE to form a compact base. It also works to stabilize FO on the other hand. This condition may lead to disparate complexation of chain packing in the matrix of the upper layer. As seen in Figure 2A1, 2B1, and 2C1, FO droplets were found on the top side of the double-layer film magnified 1000 times. It seems that the droplets were protected by a layer formed by the MG complex only or MG complex-GLE. Disintegration of the film surface due to cavities and troughs can also be caused by the bubbles since the homogenization and sonication were not performed for the upper layer formulation. Interestingly, the bottom layers of all double-layer films (1000 × magnification) possessed better uniformity, and neither holes nor troughs were observed ( Figure 2A2, 2B2, and 2C2).

### Thermal properties of double-layer edible film

**Figure 3.**
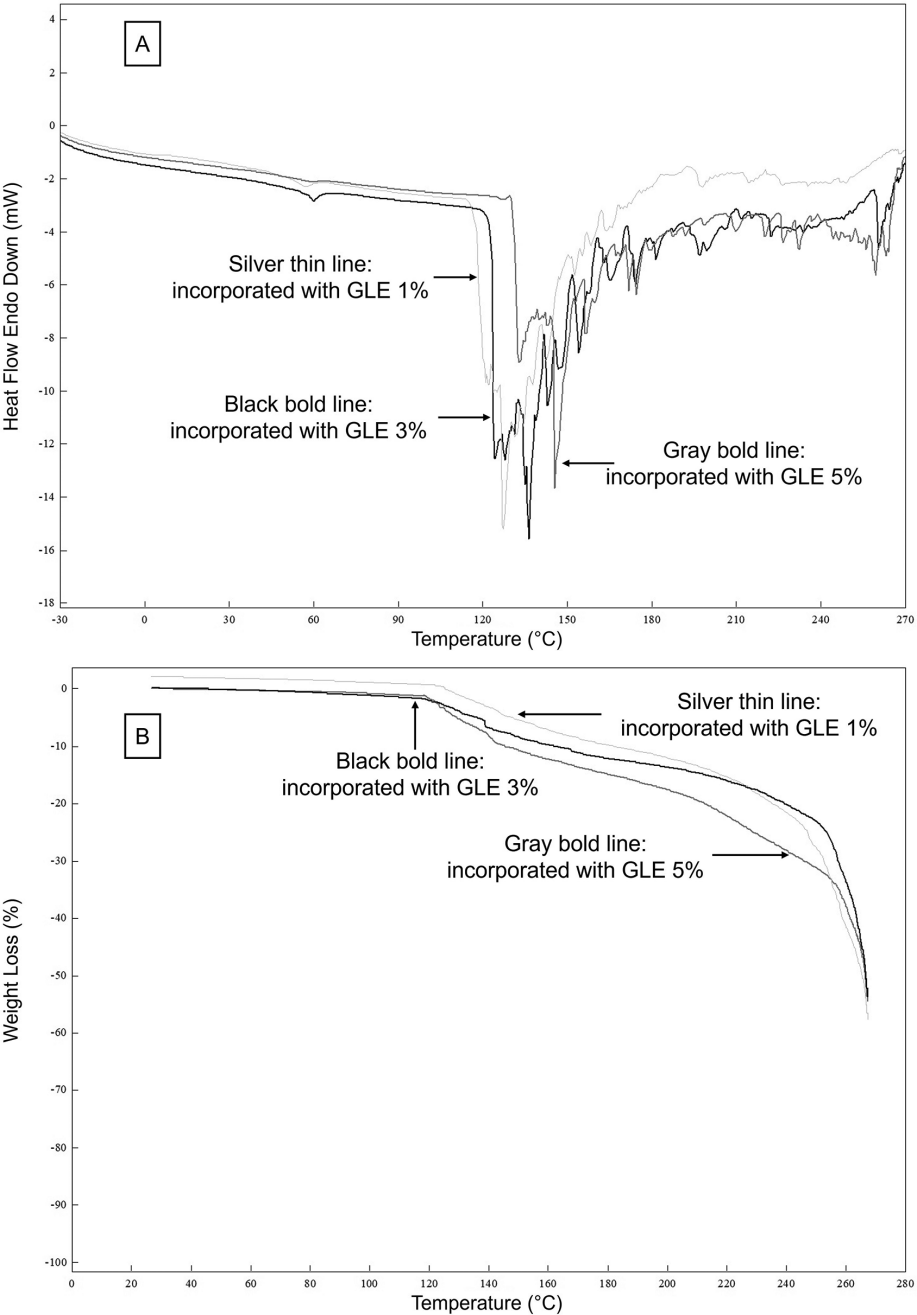
DSC (A) and TGA (B) curves of double-layer films with GLE at different addition levels. GLE 1% (silver thin line), GLE 3% (black bold line), and GLE 5% (gray bold line).

DSC scans of double-layer films incorporated with 1, 3, and 5% GLE are illustrated in Figure 3A. For DSC analysis, Table 3 shows that MGFO-GLE 3% and MGFO-GLE 5% underwent glass transition at slightly higher temperatures, 53.46°C and 57.50°C, compared to that of MGFO-GLE 1% (T1/Tg = 52.74°C). The former films were also followed by the elevated melting temperatures (T2/Tm), 142.35°C and 139.18°C respectively for MGFO-GLE 3% and MGFO-GLE 5%, whereas Tm of MGFO-GLE 1% was 131.59°C. However, the enthalpy (∆H) among the samples did not show a certain trend, and the decomposition temperatures (T3) were identical.

**Table 3.** DSC and TGA of double-layer films containing GLE at different addition levels.

Film type	DSC				TGA			
	T1 (°C)	T2 (°C)	T3 (°C)	A (mJ/mg)	WL1 (%)	WL2 (%)	WL3 (%)	WLep (%)
MGFO-GLE 1%	52.74 ± 0.24	131.59 ± 6.58	260.61 ± 0.37	259.98 ± 17.95	0.19 ± 0.04	2.39 ± 0.61	44.38 ± 2.96	57.84 ± 0.27
MGFO-GLE 3%	53.46 ± 0.61	142.35 ± 4.48	260.25 ± 1.19	247.33 ± 30.14	0.17 ± 0.10	7.94 ± 1.69	36.28 ± 1.91	56.13 ± 3.27
MGFO-GLE 5%	57.50 ± 1.17	139.18 ± 4.28	260.76 ± 0.36	266.00 ± 25.90	0.37 ± 0.05	8.51 ± 2.28	37.16 ± 0.51	53.88 ± 0.87

T1, T2, and T3 are the temperatures (°C) from DSC analysis corresponding to the glass transition, melting, and degradation of compounds, respectively. A is the DSC peak area (mJ/mg), which represents the endothermic process of melting. WL1, WL2, and WL3 are the weight losses (%) from the TGA analysis corresponding to T1, T2, and T3, respectively. WLep is the weight loss (%) at the end point. MGFO-GLE 1%: FO-containing edible films double layered with a film containing GLE 1%, MGFO-GLE 3%: FO-containing edible films double-layered with a film containing GLE 3%, and MGFO-GLE 5%: FO-containing edible films double layered with a film containing GLE 5%. Values are presented as the mean ± standard deviation (n = 2).

Our result is in contrast to previous studies that reported that the decline in Tg and Tm of edible films was caused by the rising addition of plant extracts, generated from guava leaf, ^44^ galangal, ^49^ and pomegranate peel, ^50^ to the film-forming solution. This can be attributed to the bioactive compounds of the extracts that promote the plasticization of the film by increasing its molecular mobility. ^51^ This study contrarily observed that Tg and Tm were shifted to higher temperatures as the level of GLE increased, indicating that molecular mobility was limited. Protein (MG complex) and phenol (GLE) interactions via hydrophobic interaction, electrostatic interaction, and hydrogen bonding can be attributed to this phenomenon. ^52^ This leads to the alteration of their original properties whilst creating more crosslinking so that a film with higher thermal stability can be made. ^53^ In addition, zein, as a protein part of the MG complex, has a distinctive behavior, particularly its exceptional stretchability and rubbery. ^54^ This enables the film to become preferentially extensible and less brittle without decreasing Tg and Tm. This was linked to the tensile strength and EAB of films with higher addition of GLE, MGFO-GLE 3%, and MGFO-GLE 5% ( Table 2). This study displayed higher Tg and EAB than zein films impregnated with vegetable oils, 47-50°C Tg and 0.93-8.08% EAB. ^55^ Moreover, this study produced a double-layer film that should be considered for comparison with other findings.

The TGA curves ( Figure 3B) demonstrated the weight loss of the double-layer films during glass transition, melting, and degradation. Weight losses of MGFO-GLE 5% in the first step/WL1 (glass transition), as well as MGFO-GLE 3% and MGFO-GLE 5% in the second step/WL2 (melting) were noticeable. In the third step/WL3 (degradation at 260°C) and endpoint/WLep (267°C), the weight loss of MGFO-GLE 1% was noticeable ( Table 3). The higher weight loss (WL1 and WL2) of MGFO-GLE 5% in the early stage could be associated with the greater moisture content ( Table 2). The better retention of MGFO-GLE 5% during decomposition could be explained by the abundance of protein-phenol crosslinks and other types of intermolecular interactions. A previous study developed a zein-based edible film incorporated with essential oil/β-cyclodextrin and enriched with extracts of dill leaves. ^56^ They pointed out several degradation steps of their edible films that involved the release of moisture, volatile compounds, and solvent at temperatures up to 150°C, dissociation of weakly bonded small functional groups from 150 to 270°C, and the removal of heat-stable compounds at temperatures higher than 270°C. Specifically, at 120-200°C, there is a degradation process of fatty acids. ^55^ This process was recorded in the TGA curves of this study and showed a downward trend ( Figure 3B). Furthermore, the WL3 (37.16%) and WLep (53.88%) of MGFO-GLE 5% were the lowest. Similar trends for the efficacies of the ethanolic extract of algae, ^57^ ficus extract, ^58^ and grape seed extract ^59^ at higher concentrations to enhance the thermal stability of the edible films have supported the TGA results of this study. Above all, the DSC and TGA results suggested that GLE could be a driving force for the thermal stability of the double-layer film containing FO.

### Microbiological properties of edible films

All edible films (single or double layer) possessed strong inhibitory activity against *B.* *subtilis*, but inhibition against *E.* *coli* was only found in double-layer films ranging from weak to moderate ( Table 4). The antibacterial activity was significantly changed by adjusting the additional level of GLE in the film formulation (P < 0.05). In previous studies, the antibacterial activities of the edible films were dependent on the concentration of GLE in the film-forming solution. ^44^^,^^60^ In this study, the film containing FO only (MGFO) exhibited strong inhibition (DIZ = 20.07 mm) against *B.* *subtilis*, but did not inhibit *E.* *coli.* The existence of the upper/active layer containing GLE contributed to (13-20 mm) to strengthen the antibacterial activity of the film against *B.* *subtilis.* The antibacterial activity (DIZ = 40.58 mm) of MGFO-GLE 5% was doubled *.* The incorporation of 5% GLE also markedly exerted the antibacterial activity of the film against *E. coli* (DIZ = 9.04 mm). Clearly, this result demonstrated that the double-layer edible film was more resistant to *B.* *subtilis* than *E.* *coli.* Previous studies have shown that eicosapentaenoic acid (EPA) and docosahexaenoic acid (DHA) are more effective in inhibiting *B.* *subtilis*, *Staphylococcus epidermidis*, and *Staphylococcus aureus* than *E.* *coli*, *Salmonella typhimurium*, and *Salmonella enteritidis.* ^61^^,^^62^

**Table 4.** DIZ of FO-containing edible films without or with GLE at different addition levels.

Film type	Diameter of inhibition zone (mm) against	
	*B.* *subtilis*	*E.* *coli*
MGFO	20.07 ± 3.66 ^c^ (strong)	0.00 ± 0.00 ^d^ (no inhibition)
MGFO-GLE 1%	33.67 ± 1.79 ^b^ (strong)	2.05 ± 0.65 ^c^ (weak)
MGFO-GLE 3%	37.89 ± 2.73 ^a^ ^b^ (strong)	3.57 ± 0.10 ^b^ (weak)
MGFO-GLE 5%	40.58 ± 0.63 ^a^ (strong)	9.04 ± 0.43 ^a^ (moderate)

MGFO: single-layer film containing FO, MGFO-GLE 1%: FO-containing edible films double-layered with a film containing GLE 1%, MGFO-GLE 3%: FO-containing edible films double-layered with a film containing GLE 3%, and MGFO-GLE 5%: FO-containing edible films double-layered with a film containing GLE 5%. Values are presented as the mean ± standard deviation (n = 3). Values with different lowercase superscripts in the same column indicate significant differences (P < 0.05).

Simplicity in the cell wall component of gram-positive bacteria enables the edible film material to easily penetrate the inner part of the bacteria. These fatty acids can concomitantly cause the rupture of bacterial cell membranes through disturbances in electron transport, enzyme production, and nutrient uptake, as well as the production of oxidation products. ^62^ Bacterial cell walls can also be destroyed by the antimicrobial compounds inherent in GLE. Gram-positive bacteria can carry 30-70% peptidoglycan, ^63^ allowing more hydroxyl groups of the phenolic compound to interact with the cell wall of *B.* *subtilis.* ^64^ This leads to the destruction of the cell wall integrity and the interchangeability of the inner component of the bacteria with edible film materials. Therefore, the single- and double-layer films dramatically impaired *B.* *subtilis.* Unfortunately, owing to the complexity of the cell wall of gram-negative bacteria, the double-layer film could not optimally eliminate the growth of *E.* *coli.* Exogenous long-chain fatty acids can be used for the synthesis of lipopolysaccharides in *E.* *coli* to construct a formidable outer membrane. The fatty acids are transported into the inner membrane using FadL, an outer membrane protein. ^65^ This confirmed that no antibacterial activity against *E. coli* was observed for the film containing FO only (MGFO). GLE was recognized for its role in improving the antibacterial activity of the double-layer film against *E. coli*, with a DIZ of GLE 100% was 12.55 mm ( Table 1).

**Figure 4.**
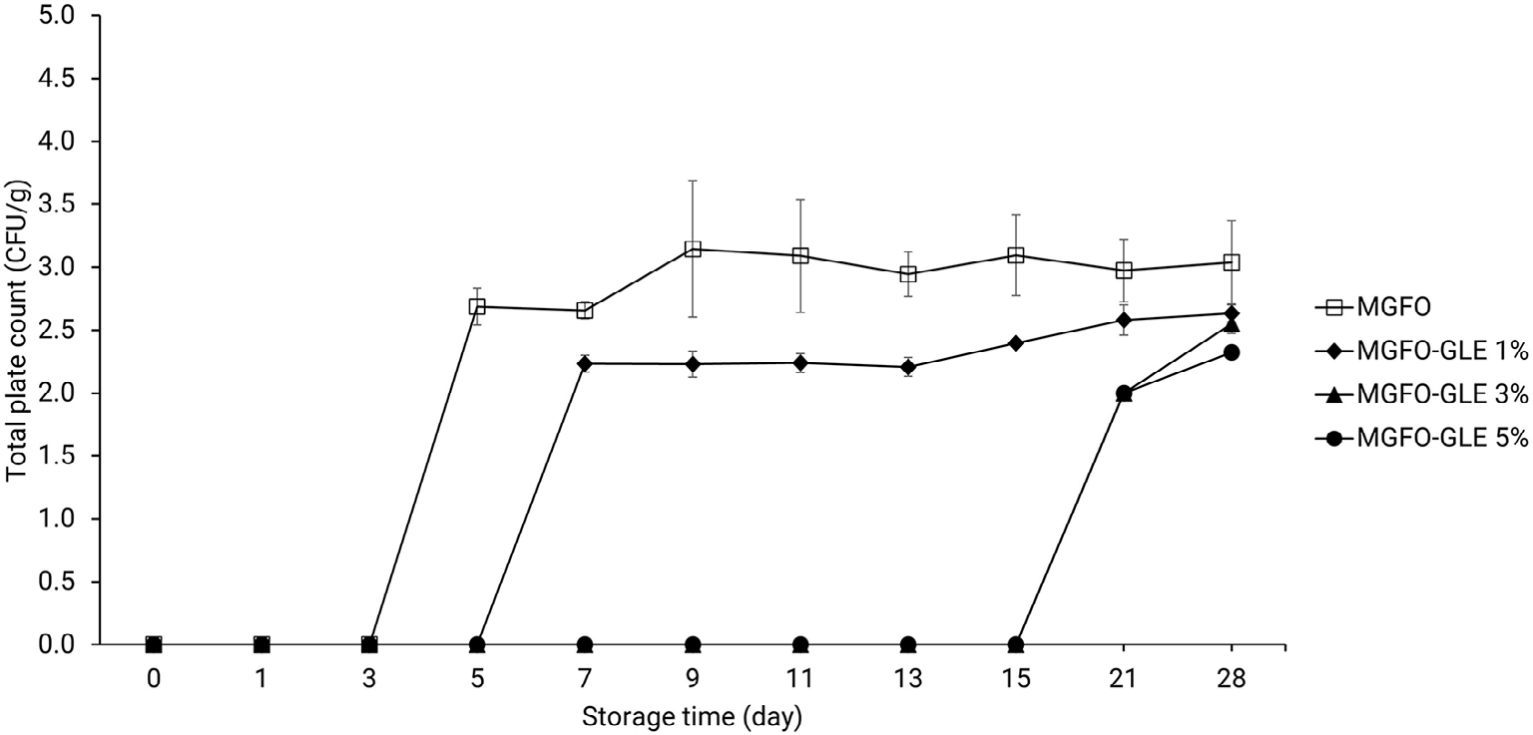
TPC of FO-containing edible films without or with GLE at different addition levels. Error bars show the standard deviation of the mean (n = 3).

According to the total plate count (TPC) in Figure 4, MGFO suppressed microbial viability until 3-day storage. MGFO-GLE 1% was found to restrain microbial viability for 5-day storage. Interestingly, microbial viability was remarkably suppressed after 15-day storage using MGFO-GLE 3% and MGFO-GLE 5%. Furthermore, the initial TPC (2 log CFU/g) of double-layer film containing higher GLE at levels of 3 and 5% was lower than the initial TPC (2.69 log CFU/g) of MGFO and TPC (2.23 log CFU/g) of MGFO-GLE 1%. Double-layer films had significantly lower TPC after 28-day storage, that is, 2.63, 2.55, and 2.32 log CFU/g for MGFO-GLE 1%, MGFO-GLE 3%, and MGFO-GLE 5%, respectively (P < 0.05). In contrast, MGFO showed higher TPC (3.04 log CFU/g) on the final day of storage. The present study found that the addition of GLE resulted in a better TPC reduction. It can also be inferred that the antibacterial activity of edible films (single- or double-layer) against *B.* *subtilis* and *E. coli* is a decisive factor in this regard.

### Peroxide value of edible films

Monitoring PV is a common means to investigate the oxidative stability of edible films. In this study, the initial PV of the single-layer edible film containing FO only (MGFO) was approximately 10.46 meq peroxide/kg sample. This amount was significantly three- to five-fold greater than the initial PV of double-layer edible film incorporated with GLE, ranging from 2.56-3.83 meq peroxide/kg sample (P < 0.05). The upward trend of the PV of MGFO gradually rocketed after 3-day storage and another surge was identified after 15-day storage. From the initial point (day 0), there was an increase in the amount of PV up to 8.76, 11.04, and 15.44 meq peroxide/kg sample on days 15, 21, and 28 of storage, respectively. A steep climbing trend in the PV of the double-layer film was also found after 15-day storage. The PV of MGFO-GLE 1% rose to just over 9 meq peroxide/kg sample on day 28 of storage. PV of MGFO-GLE 3% and MGFO-GLE 5% increased at about 6.58 and 5.31 meq peroxide/kg sample respectively, on day 28 of storage. Our results were comparable to those of a previous study that employed rosemary and oregano essential oils to prevent oxidation of FO-containing edible films. ^17^

**Figure 5.**
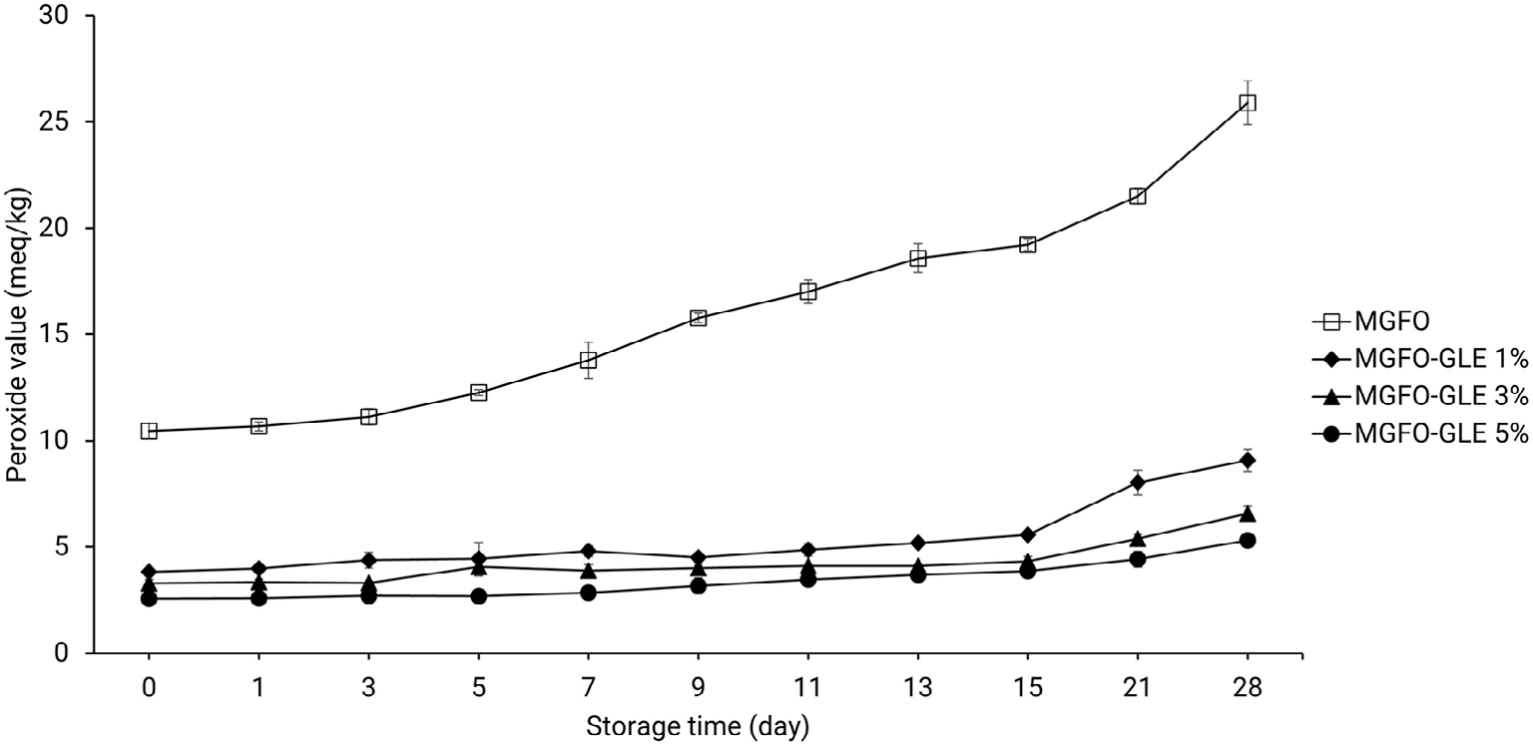
PV of FO-containing edible films without or with GLE at different addition levels. Error bars show the standard deviation of the mean (n = 3).

Although the upper layer of the double-layer film showed numerous cavities and troughs ( Figures 2A, 2B, and 2C) and the possibility of water vapor permeation ( Table 2), FO may have been stabilized by the MG complex alone or in interaction with GLE. Moreover, owing to the antioxidant activity of GLE ( Table 1), it is not surprising that its incorporation into the upper/active layer considerably controlled the oxidative stability of the double-layer edible film, either during production or storage time. Donations of hydrogen atoms and/or single electrons to free radicals are possible mechanisms by which plant extracts slow down the rate of hydroperoxide formation. ^20^^–^^22^ Based on the TPC ( Figure 4) and PV ( Figure 5) of double-layer films, it seems that the antibacterial and antioxidant activities of GLE were highly diminished after 15-day storage, indicating the soar trends in these results.

## Conclusion

The present study revealed that the incorporation of GLE at all additional levels (1, 3, and 5%) demonstrated promising improvements in the antibacterial activity and oxidative stability of double-layer edible films containing FO. At additional levels of 3 and 5%, even though the thickness of the double-layer films increased, GLE reduced the moisture barrier ability as the moisture content increased. In addition, at the same levels, GLE rendered a rugged structure that was porous on the top side of the double-layer film under SEM observation. However, GLE at these levels advantageously improved the EAB of the double-layer films while decreasing the tensile strength without lowering Tg and Tm. Other than GLE, the roles of MG complex and FO could have critical impacts on the surface integrity, thermal stability, antibacterial activity, and oxidative stability of the film. Therefore, future work will address the drawbacks of this study by appropriately adjusting the ratio of the film-forming mixture for the bottom and upper layers. The current approach will contribute to the development of alternative techniques for FO enrichment of various solid food products, such as ready-to-eat sausage, cheese, candy, and snack bars.

## Ethics and consent

Ethical approval and consent were not required.

## Data Availability

Figshare: Fish oil-containing edible films with active film incorporated with extract of *Psidium guajava* leaves: preparation and characterization of double-layered edible film. https://doi.org/10.6084/m9.figshare.26076712. ^66^ The project contains the following underlying data:
-
Figures (photograph of double-layer edible film, micrograph of double-layer edible film, DSC and TGA curves of double-layer edible film, TPC of single- and double-layer edible films, PV of single- and double-layer edible films). - Raw data of GLE (total phenolic compound, total flavonoid compound, antioxidant activity, and diameter of inhibition zone) in Microsoft Excel Worksheet. - Raw data of edible films (moisture content, thickness, WVTR, mechanical properties, DSC, TGA, diameter of inhibition zone, TPC, and PV) in Microsoft Excel Worksheet. Data are available under the terms of the Creative Commons Attribution 4.0 International license (CC-BY 4.0).
