# Supplementary material for: Fish Oil-Containing Edible Films with Active Film Incorporated with Extract of Psidium Guajava Leaves: Preparation and Characterization of Double-Layered Edible Film
Source: F1000Res. 2026 Aug 10;13:816. Originally published 2024 Jul 19. [Version 3] doi: 10.12688/f1000research.153383.3 (PMC11544199; doi:10.12688/f1000research.153383.3)
Supplement: Peer review history

# Reviewer report: Reviewer response for version 2

**DOI:** 10.5256/f1000research.173681.r336517

**Published:** 2024-12-20

**Authors:** Nurul Saadah Said

**Affiliations:** 1 Kyungpook National University, Daegu, South Korea

I have carefully reviewed the revised manuscript and the authors' responses to the previous comments. I am pleased to note that the authors have thoroughly addressed all the concerns and suggestions raised during the initial review process. Given the comprehensive of the revisions and the overall quality of the work, I believe the manuscript now meets the standards for publication. Therefore, I recommend that this manuscript be accepted for indexing in its current form.

Is the work clearly and accurately presented and does it cite the current literature?

Yes

If applicable, is the statistical analysis and its interpretation appropriate?

Yes

Are all the source data underlying the results available to ensure full reproducibility?

Yes

Is the study design appropriate and is the work technically sound?

Partly

Are the conclusions drawn adequately supported by the results?

Partly

Are sufficient details of methods and analysis provided to allow replication by others?

Yes

Reviewer Expertise:

Food packaging, film, pectin, gelatin

I confirm that I have read this submission and believe that I have an appropriate level of expertise to confirm that it is of an acceptable scientific standard.

# Reviewer report: Reviewer response for version 2

**DOI:** 10.5256/f1000research.173681.r336516

**Published:** 2024-11-08

**Authors:** Alfredo Vázquez-Ovando

**Affiliations:** 1 Instituto de Biociencias, Universidad Autonoma de Chiapas, Tapachula, Chiapas, Mexico

The modifications made by the authors allow for a highly indexable version of the manuscript.

Is the work clearly and accurately presented and does it cite the current literature?

Partly

If applicable, is the statistical analysis and its interpretation appropriate?

Yes

Are all the source data underlying the results available to ensure full reproducibility?

Yes

Is the study design appropriate and is the work technically sound?

Yes

Are the conclusions drawn adequately supported by the results?

Partly

Are sufficient details of methods and analysis provided to allow replication by others?

Yes

Reviewer Expertise:

Food science and technology, biotechnology

I confirm that I have read this submission and believe that I have an appropriate level of expertise to confirm that it is of an acceptable scientific standard.

# Reviewer report: Reviewer response for version 2

**DOI:** 10.5256/f1000research.173681.r336518

**Published:** 2024-11-07

**Authors:** Mohammed Sabbah

**Affiliations:** 1 Nutrition and Food Technology, An-Najah National University, Nablus, Palestinian Territory

Dears

 No further comments to make.

 Regards,

Is the work clearly and accurately presented and does it cite the current literature?

Partly

If applicable, is the statistical analysis and its interpretation appropriate?

Yes

Are all the source data underlying the results available to ensure full reproducibility?

Yes

Is the study design appropriate and is the work technically sound?

Yes

Are the conclusions drawn adequately supported by the results?

Yes

Are sufficient details of methods and analysis provided to allow replication by others?

Yes

Reviewer Expertise:

food edible packaging.

I confirm that I have read this submission and believe that I have an appropriate level of expertise to confirm that it is of an acceptable scientific standard.

# Reviewer report: Reviewer response for version 1

**DOI:** 10.5256/f1000research.168273.r314153

**Published:** 2024-10-21

**Authors:** Alfredo Vázquez-Ovando

**Affiliations:** 1 Instituto de Biociencias, Universidad Autonoma de Chiapas, Tapachula, Chiapas, Mexico

I think the manuscript reports an interesting study, with a good methodological approach, relevant results and regularly well written.

 I note here some slight observations to the authors

 An interesting question that arises from this approach is what happens if all the components (fish oil and guava leaf extract) are mixed in a single layer?

 If possible, that would be a good Control

 A brief information about the zein modification process should be included in the introduction

 The microbiological characterization test against pathogens in the films should be included in the methods section; that is, include without too many details the size and pretreatment given to the film before being placed on the plates with the pathogen. Remove from where it is currently mentioned (analysis of diameter of inhibition zone).

 The discussion mentions that the fish oil drops could have created cavities on the surface of the dried film. Was emulsification not performed during the manufacturing process? Is it possible that it is gas? Or where did the micelle go?

 How long did it take from the film production to the initial peroxide index measurement? Explain why the film without guava leaf extract initially had a higher value than the others. Perhaps oxidation occurred during production.

Is the work clearly and accurately presented and does it cite the current literature?

Partly

If applicable, is the statistical analysis and its interpretation appropriate?

Yes

Are all the source data underlying the results available to ensure full reproducibility?

Yes

Is the study design appropriate and is the work technically sound?

Yes

Are the conclusions drawn adequately supported by the results?

Partly

Are sufficient details of methods and analysis provided to allow replication by others?

Yes

Reviewer Expertise:

Food science and technology, biotechnology

I confirm that I have read this submission and believe that I have an appropriate level of expertise to confirm that it is of an acceptable scientific standard, however I have significant reservations, as outlined above.

# Response

**Published:** 2024-10-24

**Authors:** Aji Sukoco

**Affiliations:** Universitas Jember, Jember, East Java, Indonesia

Dear reviewer,

 We sincerely apologize for the delay in responding to your valuable comments on our paper. We are grateful for the opportunity to improve the quality of our paper entitled “ **Fish oil-containing edible films with active film incorporated with extract of *Psidium guajava* leaves: preparation and characterization of double-layered edible film**”. We have meticulously read the comments from the reviewer. We hope that the comments can be addressed by our additional explanations below. Thank you again for your great support by giving us constructive comments and suggestions.

 Sincerely yours,

 Authors: Aji Sukoco, Yukihiro Yamamoto*, Hiroyuki Harada, Atsushi Hashimoto, and Tomoyuki Yoshino

 Reviewer comments and author responses

**Comment 1:**

 An interesting question that arises from this approach is what happens if all the components (fish oil and guava leaf extract) are mixed in a single layer?

 If possible, that would be a good Control.

**Response to reviewer’s comment 1:**

 Thank you, it is an interesting question. Based on the findings of the high peroxide value of MGFO (single-layer film containing FO), we decided to use GLE in this study. We made the film with GLE in a double-layer structure as we thought that this concept could be better to completely protect the FO by considering the different characteristics of each layer, hydrophobic for the lower layer (containing FO) and more hydrophilic for the upper layer (containing GLE). As an edible film is usually placed on the foods in its actual application, which is highly in contact with the surroundings, the placement of FO-containing film in between the food matrix and GLE-containing film can be a good strategy to minimize the rapid oxidation of FO. In our study, the result of the peroxide values of MGFO and MGFO-GLE is shown in Figure 5. So, we guess that a single-layer film containing both FO and GLE may result in a high peroxide value because FO will be directly exposed to the environment. GLE and FO stand individually in the film matrix due to their different characteristics that cannot be easily managed by the MG complex.

**Comment 2:**

 A brief information about the zein modification process should be included in the introduction.

**Response to reviewer’s comment 2:**

 Thank you for your valuable suggestion. We have emphasized our sentences in the “Preparation of modified zein-gum arabic complex” section with respect to your concerns. We have added an explanation in the last paragraph of the introduction section: *We used glycerol and sodium dodecyl sulfate (SDS) to prevent the aggregation of zein particles*.

**Comment 3:**

 The microbiological characterization test against pathogens in the films should be included in the methods section; that is, include without too many details the size and pretreatment given to the film before being placed on the plates with the pathogen. Remove from where it is currently mentioned (analysis of diameter of inhibition zone).

**Response to reviewer’s comment 3:**

 Thank you for your suggestion. As per the instruction of F1000Research, *the Methods sections should provide sufficient details of the materials and methods used so that the work can be repeated by others*, we explained this section accordingly.

**Comment 4:**

 The discussion mentions that the fish oil drops could have created cavities on the surface of the dried film. Was emulsification not performed during the manufacturing process? Is it possible that it is gas? Or where did the micelle go?

**Response to reviewer’s comment 4:**

 Thank you for your deep review of this issue, it could enrich the information on our paper. Homogenization and sonication can sufficiently generate a fine emulsion, and this was only done for the lower layer formulation (containing FO). We did not perform such processes for the upper layer formulation (containing GLE) to prevent adverse effects on the chemical and/or structural properties of GLE. We agree with your opinion, so we have added an explanation in the second paragraph of the "Morphology of double-layer edible film" section: *Disintegration of the film surface due to cavities and troughs can also be caused by the bubbles since the homogenization and sonication were not performed for the upper layer formulation*.

**Comment 5:**

 How long did it take from the film production to the initial peroxide index measurement? Explain why the film without guava leaf extract initially had a higher value than the others. Perhaps oxidation occurred during production.

**Response to reviewer’s comment 5:**

 Thank you for your question. Measurement of the initial (day 0) peroxide value was done using freshly prepared films. We need approximately 60 min to cool down the temperature of the film before peeling. MGFO (single-layer film containing FO) was made after the 48-h drying process, while MGFO-GLE (double-layer) was made after the 96-h drying process. In the MGFO sample, FO can be rapidly oxidized during drying and cooling. However, FO oxidation of MGFO-GLE can still be retarded during the prolonged production time due to a layer containing GLE that has an antioxidative effect. We have emphasized our sentences in the second paragraph of the "Peroxide value of edible films" section with respect to your concerns: *Moreover, owing to the antioxidant activity of GLE ( Table 1), it is not surprising that its incorporation into the upper/active layer considerably controlled the oxidative stability of the double-layer edible film, either during production or storage time. Donations of hydrogen atoms and/or single electrons to free radicals are possible mechanisms by which plant extracts slow down the rate of hydroperoxide formation.*

# Reviewer report: Reviewer response for version 1

**DOI:** 10.5256/f1000research.168273.r317481

**Published:** 2024-09-18

**Authors:** Nurul Saadah Said

**Affiliations:** 1 Kyungpook National University, Daegu, South Korea

**After reviewing the manuscript entitled 'Fish oil-containing edible films with active film incorporated with extract of Psidium guajava leaves: preparation and characterization of double-layered edible film', I found that this publication can be accepted for indexing after some major revisions. The comments are as follows:**

1. The English needs to be checked, especially the grammar and word choice in the introduction and abstract sections.
2. What is the significance of mixing zein, fish oil, and GLE as different layered film structures?
3. On page 6, under characteristics of GLE, it was mentioned that 100% GLE showed a powerful antibacterial effect against tested bacteria, and it was selected as the compound for the upper layer film. However, in the film formulation, the GLE incorporated into the film was in the range of 1-5%. Please clarify this discrepancy.
4. The WVTR analysis should discuss factors based on both sides of the film since it is double-layered. The functions of zein and fish oil should be discussed, especially zein, as it was mentioned in the introduction to impart hydrophobicity to the films.
5. Please explain further why film extensibility (EAB) increases with higher GLE concentration. Please provide the trends results and references from previous studies as well.
6. Please correlate the SEM structure with WVTR and mechanical properties results, as the uneven surface or open pores could be factors that impact the film properties.
7. For the discussion on microbiological properties of films, how can you confirm that the antibacterial properties are solely attributed to GLE? In Table 1, GLE within 25-75% showed weak to moderate antibacterial activity. However, in the film, only 3-5% of GLE was incorporated and showed strong antibacterial activity, contradicting the findings in Table 1. Please clarify whether other factors such as zein or fish oil could be contributing to this significant effect.
8. Please provide photos of the plate count for better understanding.
9. In most discussions, only GLE was being discussed, while the functions of fish oil, gum, and zein were not highlighted in the abstract and conclusion. Please rewrite these sections, as other materials could contribute to the film properties.

Is the work clearly and accurately presented and does it cite the current literature?

Yes

If applicable, is the statistical analysis and its interpretation appropriate?

Yes

Are all the source data underlying the results available to ensure full reproducibility?

Yes

Is the study design appropriate and is the work technically sound?

Partly

Are the conclusions drawn adequately supported by the results?

Partly

Are sufficient details of methods and analysis provided to allow replication by others?

Yes

Reviewer Expertise:

Food packaging, film, pectin, gelatin

I confirm that I have read this submission and believe that I have an appropriate level of expertise to confirm that it is of an acceptable scientific standard, however I have significant reservations, as outlined above.

# Response

**Published:** 2024-10-24

**Authors:** Aji Sukoco

**Affiliations:** Universitas Jember, Jember, East Java, Indonesia

Dear reviewer,

 We sincerely apologize for the delay in responding to your valuable comments on our paper. We are grateful for the opportunity to improve the quality of our paper entitled “ **Fish oil-containing edible films with active film incorporated with extract of *Psidium guajava* leaves: preparation and characterization of double-layered edible film**”. We have meticulously read the comments from the reviewer. We hope that the comments can be addressed by our additional explanations below. Thank you again for your great support by giving us constructive comments and suggestions.

 Sincerely yours,

 Authors: Aji Sukoco, Yukihiro Yamamoto*, Hiroyuki Harada, Atsushi Hashimoto, and Tomoyuki Yoshino

 Reviewer comments and author responses

**Comment 1:**

 The English needs to be checked, especially the grammar and word choice in the introduction and abstract sections.

**Response to reviewer’s comment 1:**

 Thank you for your suggestion. Before being published, our paper was checked by an academic writing tool provided by F1000Research. We have revised the language and grammar of our paper accordingly. In the abstract section, we have changed the word “shelter” to “delivery system”.

**Comment 2:**

 What is the significance of mixing zein, fish oil, and GLE as different layered film structures?

**Response to reviewer’s comment 2:**

 Thank you for the comment. We have emphasized our sentences in the introduction section with respect to your concerns. We have elaborated it point-by-point, starting with the main challenge of FO for food uses which is associated with its oxidation rate (first paragraph). Next, the edible film is one of the alternatives to carrying FO without being involved in food processing. The use of single-layered edible film still poses a risk to the oxidative stability of FO, even if the antioxidative agent is incorporated. Therefore, a double-layered edible film is studied (second paragraph). After that, GLE is incorporated in an outer/upper layer with no FO to completely protect the FO-containing layer (bottom/lower layer). The advantage of GLE is also mentioned for both antioxidant and antimicrobial boosters (third paragraph). Following this, zein and GA are selected biopolymers to build a wall material for FO and GLE, as well as to construct a robust film matrix (fourth paragraph).

**Comment 3:**

 On page 6, under characteristics of GLE, it was mentioned that 100% GLE showed a powerful antibacterial effect against tested bacteria, and it was selected as the compound for the upper layer film. However, in the film formulation, the GLE incorporated into the film was in the range of 1-5%. Please clarify this discrepancy.

**Response to reviewer’s comment 3:**

 Thank you, we sincerely appreciate your deep review. Before being incorporated into the film formulation, the antibacterial activity of GLE was tested at concentrations of 25-100%. It was found that GLE 100% exhibited the strongest antibacterial effects against *E*. *coli* and *B*. *subtilis*. Next, this GLE 100% (with no dilution) was incorporated into film formulation at additional levels of 1, 3, and 5% (w/v of the complex). So, 100% is the concentration of GLE, while 1-5% is the additional level of GLE 100% in the film formulation.

**Comment 4:**

 The WVTR analysis should discuss factors based on both sides of the film since it is double-layered. The functions of zein and fish oil should be discussed, especially zein, as it was mentioned in the introduction to impart hydrophobicity to the films.

**Response to reviewer’s comment 4:**

 Thank you, we sincerely appreciate your deep review. In this paper, we focused on the characterization of double-layered edible film as affected by the different additional levels of GLE. MGFO (single-layer film containing FO) was only tested for the discussion in microbiological and peroxide value sections to prove the main function of GLE, as antioxidant and antimicrobial agents. We agree with your opinion, therefore, MGFO can be tested for further evaluation in future research projects to gain a comprehensive understanding of single- and double-layered edible films containing FO. We have added some explanations in the “Physical and mechanical properties of double-layer edible film” section: *Additionally, the lower layer without GLE can be responsible for the reinforced water vapor barrier with respect to the hydrophobicity of zein and FO. Therefore, further characterizations of the physical properties of MGFO are needed to gain a comprehensive understanding of single- and double-layered edible films*.

**Comment 5:**

 Please explain further why film extensibility (EAB) increases with higher GLE concentration. Please provide the trends results and references from previous studies as well.

**Response to reviewer’s comment 5:**

 Thank you for the comment and suggestion. We have emphasized our sentences in the second paragraph of the “Physical and mechanical properties of double-layer edible film” section with respect to your concerns. However, due to the limited existing studies about FO-containing edible film without or with antimicrobial/antioxidative agents, it is difficult to make a wide comparison with our results. We have added some explanations in this section: *Instead, thicker films of MGFO-GLE 3% and MGFO-GLE 5% were less rigid and had higher extensibility due to stronger plasticizing effects and higher moisture content. The interaction between phenolic compounds and biopolymers may also have restricted the formation of intermolecular hydrogen bonds to exert a structural relaxation of the film. Previous findings found a lower EAB (less than 15%) with a higher tensile strength (less than 0.8 MPa) for FO-containing edible film enriched with rosemary and oregano essential oils. ^17^ The types of natural active substances and biopolymers can impart distinctive effects on the mechanical aspect of edible films due to chemical bonds and structural formation between them*.

**Comment 6:**

 Please correlate the SEM structure with WVTR and mechanical properties results, as the uneven surface or open pores could be factors that impact the film properties.

**Response to reviewer’s comment 6:**

 Thank you for your interest in this issue. We have emphasized our sentences about the correlation between SEM structure and WVTR in the first paragraph of the “Morphology of double-layer edible film” section. We have also added some explanations in the first paragraph of this section: *The porous surface directly permits water vapor transfer from the external or internal surroundings through the film matrix. Additionally, uneven surfaces with large cavities and troughs have made MGFO-GLE 3% and MGFO-GLE 5% less resistant (easy to break) to applying tensile strength. It was indicated by lower tensile strength values but higher EAB values, at once, confirming that they were inversely proportional relationships*.

**Comment 7:**

 For the discussion on microbiological properties of films, how can you confirm that the antibacterial properties are solely attributed to GLE? In Table 1, GLE within 25-75% showed weak to moderate antibacterial activity. However, in the film, only 3-5% of GLE was incorporated and showed strong antibacterial activity, contradicting the findings in Table 1. Please clarify whether other factors such as zein or fish oil could be contributing to this significant effect.

**Response to reviewer’s comment 7:**

 We appreciate your deep evaluation of the microbiological aspect of edible film. We would like to confirm that:

- Table 1 shows the antibacterial activity of GLE 100% (without dilution) and GLE 25-75% (with dilution).
- Table 4 shows the antibacterial activity of edible film incorporated with GLE at a concentration of 100%, which was added into film formulation at additional levels of 1, 3, and 5%. We did not use GLE at concentrations of 25-75% in the preparation of edible film.

For the analysis of the antibacterial activity of GLE 100%, it was shown that its effect was moderate against *B*. *subtilis* and strong against *E*. *coli*. When GLE 100% was incorporated into film formulation at all additional levels (1-5%), the film demonstrated a strong inhibition effect against *B*. *subtilis*. This was not only caused by GLE since MGFO (film without GLE) had a strong inhibition, which indicated that FO played an important role in this regard. The effectivity of the component in FO has been mentioned in the “Microbiological properties of edible films” section, such as in the last sentence in the first paragraph and the second sentence in the second paragraph. We have also revised the first paragraph in this section: *The antibacterial activity was significantly changed by adjusting the additional level of GLE in the film formulation (P < 0.05). In previous studies, the antibacterial activities of the edible films were dependent on the concentration of GLE in the film-forming solution. ^42 , 58^* The antibacterial activity of the edible film against *E*. *coli* has been detailed in the second paragraph of this section.

**Comment 8:**

 Please provide photos of the plate count for better understanding.

**Response to reviewer’s comment 8:**

 Thank you for your suggestion. We have uploaded the photos related to TPC analysis to the data repository. However, we only have limited documentation since we did not anticipate making complete documentation for this analysis. Again, thank you for noting this and we hope the photo is sufficient to represent our activity in this analysis.

**Comment 9:**

 In most discussions, only GLE was being discussed, while the functions of fish oil, gum, and zein were not highlighted in the abstract and conclusion. Please rewrite these sections, as other materials could contribute to the film properties.

**Response to reviewer’s comment 9:**

 Thank you for your interest in this part. Referring to the purpose of this study: *This study was undertaken to characterize FO-containing edible films that were double-layered with a film containing GLE*, we thought that GLE was the key driving force in rendering the beneficial and detrimental effects on the characteristics of edible film because it was added at different additional levels. On the other hand, MG complex and FO were added at the same amount in the film formulation. We agree with your opinion, they still have a contribution to the film properties. We have added an explanation in the last sentence in the abstract section: *MG complex and FO can also contribute to the performance of the edible film*. We have added an explanation in the conclusion section: *Other than GLE, the roles of MG complex and FO could have critical impacts on the surface integrity, thermal stability, antibacterial activity, and oxidative stability of the film*.

# Reviewer report: Reviewer response for version 1

**DOI:** 10.5256/f1000research.168273.r317480

**Published:** 2024-09-10

**Authors:** Mohammed Sabbah

**Affiliations:** 1 Nutrition and Food Technology, An-Najah National University, Nablus, Palestinian Territory

The manuscript entitled "Fish oil-containing edible films with active film incorporated with extract of *Psidium guajava* leaves: preparation and characterization of double-layered edible film". Overall, this article presents a well-structured investigation into double-layered edible films containing fish oil and guava leaf extract. Here are some specific comments and suggestions:

1. I suggest in the part of the Preparation of guava leaf extract to cite the reference of the method used. I recommend to see this paper Ref 1. 
2. I suggest citing the method that was used in the part of the analysis of the diameter of the inhibition zone.
3. While the double-layered film concept is interesting, further discussion on the novelty compared to existing research on fish oil-containing edible films is needed.
4. The rationale behind the specific concentrations used for fish oil (5%) and guava leaf extract (1, 3, and 5%) could be explained in more detail. Were these chosen based on preliminary experiments or existing literature?
5. The conclusion could be strengthened by emphasizing the potential applications of these double-layered films in food packaging.

Regards.

Is the work clearly and accurately presented and does it cite the current literature?

Partly

If applicable, is the statistical analysis and its interpretation appropriate?

Yes

Are all the source data underlying the results available to ensure full reproducibility?

Yes

Is the study design appropriate and is the work technically sound?

Yes

Are the conclusions drawn adequately supported by the results?

Yes

Are sufficient details of methods and analysis provided to allow replication by others?

Yes

Reviewer Expertise:

food edible packaging.

I confirm that I have read this submission and believe that I have an appropriate level of expertise to confirm that it is of an acceptable scientific standard, however I have significant reservations, as outlined above.

## References

[1] : Production and Characterization of Active Pectin Films with Olive or Guava Leaf Extract Used as Soluble Sachets for Chicken Stock Powder. *Coatings* .2023;13(7) : 10.3390/coatings13071253 10.3390/coatings13071253

# Response

**Published:** 2024-10-24

**Authors:** Aji Sukoco

**Affiliations:** Universitas Jember, Jember, East Java, Indonesia

Dear reviewer,

 We sincerely apologize for the delay in responding to your valuable comments on our paper. We are grateful for the opportunity to improve the quality of our paper entitled “ **Fish oil-containing edible films with active film incorporated with extract of *Psidium guajava* leaves: preparation and characterization of double-layered edible film**”. We have meticulously read the comments from the reviewer. We hope that the comments can be addressed by our additional explanations below. Thank you again for your great support by giving us constructive comments and suggestions.

 Sincerely yours,

 Authors: Aji Sukoco, Yukihiro Yamamoto*, Hiroyuki Harada, Atsushi Hashimoto, and Tomoyuki Yoshino

 Reviewer comments and author responses

**Comment 1: **I suggest in the part of the Preparation of guava leaf extract to cite the reference of the method used. I recommend to see this paper Ref 1.

**Response to reviewer’s comment 1: **Thank you for your valuable suggestion. We have added the reference (Sabbah *et al*., 2023) related to the preparation of GLE.

**Comment 2: **I suggest citing the method that was used in the part of the analysis of the diameter of the inhibition zone.

**Response to reviewer’s comment 2: **Thank you for your suggestion. We have added the reference (Mostafa *et al*., 2018) related to the analysis of the diameter of the inhibition zone.

**Comment 3: **While the double-layered film concept is interesting, further discussion on the novelty compared to existing research on fish oil-containing edible films is needed.

**Response to reviewer’s comment 3: **Thank you for your positive feedback and suggestions. To our knowledge, FO-containing edible film without or with antimicrobial/antioxidative agents is still rarely studied. This becomes a limitation in making a wide comparison with our results. However, it is noteworthy to note that our current result could tackle the main challenge of utilizing FO in edible film formulation, which is oxidation. It can be slowed down by the presence of a GLE-containing upper layer, as shown in Figure 5. We have made a comparison statement to the previous study of Duan *et al*. (2011) which can be found in the last sentence in the first paragraph of the “Peroxide value of edible films” section. Therefore, we believe that our study can be a piece of useful information for constructing a comprehensive discussion in the future.

**Comment 4: **The rationale behind the specific concentrations used for fish oil (5%) and guava leaf extract (1, 3, and 5%) could be explained in more detail. Were these chosen based on preliminary experiments or existing literature?

**Response to reviewer’s comment 4: **Thank you for the suggestions and comments. These concentrations were selected based on our unpublished reports. For the selection of FO concentration, 5, 10, and 15% of FO were incorporated into the edible film formulation. Improved characteristics in structural, thermal, and oxidative stability aspects of the edible film were properly achieved when using FO 5%. However, the initial point of peroxide value of the edible film was still relatively high up to 10 meq/kg, as shown in Figure 5. To deal with this, GLE at a concentration of 100% and at various additional levels (0.25, 0.5, 0.75, and 1%) in the film formulation was previously tested. Unfortunately, they did not show a significant reduction in peroxide value and also an improvement in antibacterial activity. So, we used a higher range of GLE levels up to 5% for further evaluation.

 We have added some explanations in our paper as suggested by the reviewer. Please find them in the first and second paragraphs of the “Preparation of double-layer edible film” section. In the first paragraph: *FO 5% was chosen based on the preliminary test in our lab. It was revealed that at a concentration of 5%, FO properly improved the characteristics of the edible film in structural, thermal, and oxidative stability aspects, compared to those of FO 10 and 15% (unpublished report)*. In the second paragraph: *GLE at an additional level of less than 1% did not significantly improve the oxidative stability and antibacterial activity of FO-containing edible film (unpublished report)*.

**Comment 5: **The conclusion could be strengthened by emphasizing the potential applications of these double-layered films in food packaging.

**Response to reviewer’s comment 5: **Thank you for the suggestion. We have added additional information in the last sentence in the paragraph of the conclusion section: *The current approach will contribute to the development of alternative techniques for FO enrichment of various solid food products, such as ready-to-eat sausage, cheese, candy, and snack bars*.
